# Case Manifestations and Public Health Response for Outbreak of Meningococcal W Disease, Central Australia, 2017

**DOI:** 10.3201/eid2607.181941

**Published:** 2020-07

**Authors:** Eva L. Sudbury, Siobhan O’Sullivan, David Lister, Deepa Varghese, Keshan Satharasinghe

**Affiliations:** Alice Springs Hospital, Alice Springs, Northern Territory, Australia

**Keywords:** case manifestations, public health response, outbreak, meningococcal W disease, invasive meningococcal disease, serogroup W, aboriginal, indigenous population, Torres Strait Islander, Neisseria meningitidis, bacteria, MenACWY vaccine, meningitis/encephalitis, Central Australia

## Abstract

*Neisseria meningitidis* serogroup W has emerged as an increasingly common cause of invasive meningococcal disease worldwide; the average case-fatality rate is 10%. In 2017, an unprecedented outbreak of serogroup W infection occurred among the Indigenous pediatric population of Central Australia; there were 24 cases over a 5-month period. Among these cases were atypical manifestations, including meningococcal pneumonia, septic arthritis, and conjunctivitis. The outbreak juxtaposed a well-resourced healthcare system against unique challenges related to covering vast distances, a socially disadvantaged population, and a disease process that was rapid and unpredictable. A coordinated clinical and public health response included investigation of and empiric treatment for 649 febrile children, provision of prophylactic antimicrobial drugs for 465 close contacts, and implementation of a quadrivalent meningococcal ACWY conjugate vaccine immunization program. The response contained the outbreak within 6 months; no deaths and only 1 case of major illness were recorded.

Invasive meningococcal disease (IMD) remains a major cause of death and permanent disability worldwide (*1*). IMD is caused by *Neisseria meningitidis*, a gram-negative diplococcus bacterium, which frequently colonizes the human nasopharynx and might spread from person-to-person by respiratory droplets or direct contact with respiratory secretions. However, only a small proportion of persons will show development of invasive infection, typically with serogroups A, B, C, W or Y (*2*). IMD is most common in the dry winter and spring, in overcrowded households, and in persons who have preceding upper respiratory tract infections, splenectomy, and in the presence of terminal complement deficiencies (*3*,*4*).

Untreated, IMD can rapidly progress to death or major disability because of sepsis and the sequelae of meningitis. Early disease identification and treatment with parenteral antimicrobial drugs are vital in reducing illness and death. Initial signs and symptoms of the disease can be nonspecific and difficult to distinguish from those of less severe illnesses (*5*). In addition to the typical manifestations of sepsis or meningitis, atypical manifestations are well described and include septic arthritis, pneumonia, pericarditis, gastroenteritis, and epiglottitis (*5*–*9*). Conjunctivitis is also recognized, although is not usually associated with systemic illness (*10*,*11*).

Since early outbreaks were described during the Hajj pilgrimages of 2000 and 2001 (*12*,*13*), IMD caused by *N. meningitidis* serogroup W (MenW) has been increasingly responsible for epidemics globally (*1*,*14*–*16*). MenW has also emerged as a cause for endemic disease in South Africa, the United Kingdom, and Chile (*17*–*19*).

In Australia, IMD has historically been caused by endemic cases of serogroup B and C infection (*3*,*6*). Since 2013, there has been an increase in the incidence and proportion of IMD caused by MenW; in 2016, it was the predominant meningococcal serogroup in Australia (*3*,*6*). During 2003–2015, the case-fatality rate for infection with MenW in Australia was 10.7%, which was more than twice the case-fatality rate for all IMD serogroups combined (*6*). Until 2017, the Northern Territory was the only state in Australia that had not experienced an increase in IMD caused by MenW (*20*).

A conjugate meningococcal C vaccine has been funded and provided by the Australian National Immunisation Program since 2003 for all children at the age of 12 months, which reflects the previous predominance of IMD caused by this serogroup (*6*,*21*). A meningococcal B vaccine, and several quadrivalent meningococcal A, C, W, Y (MenACWY) conjugate vaccines, are registered for use in Australia but were not part of the funded program during 2017 (*6*,*21*).

During July–December 2017, an outbreak of IMD caused by MenW occurred in Central Australia. We report case manifestations and the clinical and public health response.

## Methods

### Ethics and Setting

**Figure 1:**
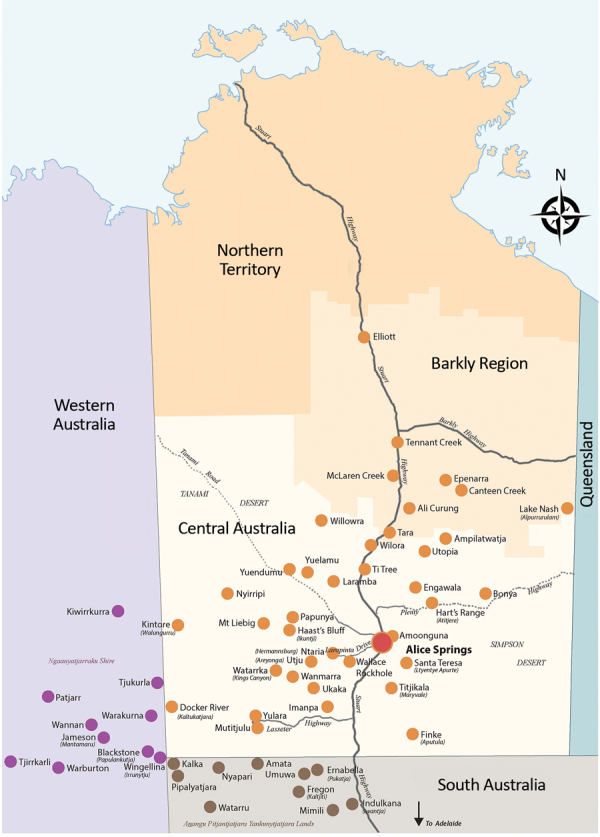
Alice Springs Hospital catchment area, Central Australia. Red dot indicates Alice Springs township; orange dots indicate Northern Territory communities; purple dots indicate Western Australia communities; gray dots indicate South Australia communities.

This study was approved by the Central Australian Human Research Ethics Committee (CA-18-3032). Alice Springs Hospital is the regional referral center for Central Australia and has a catchment area that covers ≈1.6 million km^2^ (Figure 1). This area encompasses the city of Alice Springs, Northern Territory, and the surrounding remote communities and has a total population of ≈60,000 persons, many of whom are Indigenous Australians, identifying as Aboriginal or Torres Strait Islander (ATSI). The hospital has a 40-bed pediatric ward with ≈1,800 pediatric admissions/year. The closest tertiary pediatric service with a pediatric intensive care unit (ICU) is ≈1,600 km away, in Adelaide, South Australia.

Of the 58 communities within the catchment area, 20 are within South Australia and Western Australia state jurisdictions. Pediatric patients from these communities are treated at Alice Springs Hospital because of cultural links and the relative proximity of the pediatric unit in comparison to those within state borders. The communities are located 80–1,000 km from the hospital. Most remote communities are serviced by a local medical clinic staffed by remote area nurses or general practitioners. The clinics are equipped with common antimicrobial drugs, including ceftriaxone, and are able to administer these drugs by intravenous cannulation or intramuscular injection. The clinic staff do not routinely perform more complex investigative procedures such as lumbar punctures and difficult venipuncture.

### Case Definitions and Laboratory Methods

After the outbreak period, we conducted a retrospective review of MenW cases treated by the Alice Springs Hospital pediatric service during July–December 2017. We used a standardized questionnaire to gather information from medical records at the hospital and, where necessary, at the community clinics. We defined cases as detection of *N. meningitidis* by culture or PCR from a usually sterile site (blood, cerebrospinal fluid [CSF], or synovial fluid) or from purulent eye discharge, with serogrouping demonstrating serogroup W.

Culture samples were initially incubated in the Bact/Alert 3D Microbial Detection System (bioMérieux, https://www.biomerieux.com). Positive blood cultures were then inoculated onto chocolate agar and further incubated in an atmosphere of 5% CO_2_ at 35°C. Colonies were identified *N. meningitidis* by using API NH (bioMérieux). Susceptibility testing was performed by using Etest (bioMérieux) and the Australian Meningococcal Surveillance Programme interpretive criteria (*20*) and serogroup determined by using Pastorex Meningitis test kits (Bio-Rad, https://www.bio-rad.com) and Remel Meningococcus Agglutinating Sera (ThermoFisher Scientific, https://www.thermofisher.com). Meningococcal PCR was used for the *sod*C, *por*A, and *ctr*A genes by using reported methods (*22*,*23*). Cultured isolates were subsequently characterized by using multilocus sequence typing (*24*).

### Clinical and Public Health Response

In September 2017, after 9 case-patients with MenW (4.1 cases/100,000 persons in the ATSI population <15 years of age) had been admitted to the pediatric ward at Alice Springs Hospital, the Centre for Disease Control (CDC) in the Northern Territory declared an outbreak (*25*). A clinical and public health response was coordinated by CDC and Alice Springs Hospital.

The team developed a clinical case definition to denote any child <16 years of age who had a fever (temperature >38°C) as a suspected case-patient with IMD. A management protocol, referred to as the fever protocol, was instituted in which all patients from the Alice Springs Hospital catchment area who fit the case definition were investigated by using a blood culture and meningococcal PCR, and treated empirically with intravenous or intramuscular ceftriaxone (100 mg/kg/day) until their results were available. Additional investigations (e.g., lumbar puncture) were performed as clinically indicated. All primary and secondary health services in the Alice Springs Hospital catchment area across the Northern Territory, South Australia, and Western Australia followed this protocol. Patients from remote communities were retrieved by the Royal Flying Doctor Service (RFDS) and admitted to Alice Springs Hospital for observation until their results were available. For patients from urban Alice Springs, access to transport and social circumstances were considered in determining admission to the pediatric ward or discharge home with outpatient review within 24 hours by a dedicated, daily, pediatric fever clinic. Cases were classified on the basis of clinical signs and symptoms at presentation and investigation results (Table 1) and based on case definitions used in previous outbreaks (*7*,*10*,*11*,*26*–*29*).

**Table 1:** Diagnostic categories of patients with meningococcal serogroup W infection, Central Australia, 2017*

Diagnostic category (reference)	Clinical signs/symptoms	Site of MenW isolation by culture or PCR	Additional investigations
Meningococcemia (*28**,**29*)	Fever (temperature >38°C) without a focus	Blood	None
Meningitis	Fever; any signs of meningism (e.g., headache, neck stiffness, photophobia)	CSF	None
Bacteremic pneumonia (*7**,**26**,**27*)	Fever; cough	Blood	Radiologic consolidation by chest radiograph
Septic arthritis (*7*)	Fever; joint pain and swelling	Synovial fluid	None
Conjunctivitis (*10**,**11*)	Conjunctival inflammation with purulent discharge	Eye discharge	None

*CSF, cerebrospinal fluid; MenW, serogroup W of *Neisseria meningitidis*.

Concurrent to the clinical response, CDC launched a widespread public health response that included providing prophylactic antimicrobial drugs (ceftriaxone or ciprofloxacin) to close contacts and implementing an MenACWY immunization campaign. A total of 530 close contacts (*30*) were identified, of whom 465 (87.7%) received prophylactic antimicrobial drugs.

The immunization campaign began in early October 2017. Initially, the vaccine was provided for all persons 1–19 years of age who were living in remote communities in the Central Australia, Barkly, and Katherine West regions. In urban Alice Springs, Tennant Creek, and Katherine, the vaccine was initially provided to ATSI persons (1–19 years of age), but on November 1, 2017, this campaign was expanded to also include non-Indigenous persons. On December 1, 2017, the MenACWY vaccine replaced the meningococcal C vaccine in the Northern Territory vaccination schedule for all babies 12 months of age. As March 21, 2018, the MenACWY vaccine had been administered to 81% of the estimated eligible ATSI population and to 49% of the estimated eligible non-Indigenous population living in the outbreak area (*31*).

As the immunization campaign was implemented, the protocol for managing febrile patients was adjusted to reflect immunization status. Management of immunized patients was based on their clinical signs and symptoms, rather than on the presumption of IMD. The outbreak was declared over on March 23, 2018, 3 months after the last case was identified.

## Results

### Outbreak Description

**Figure 2:**
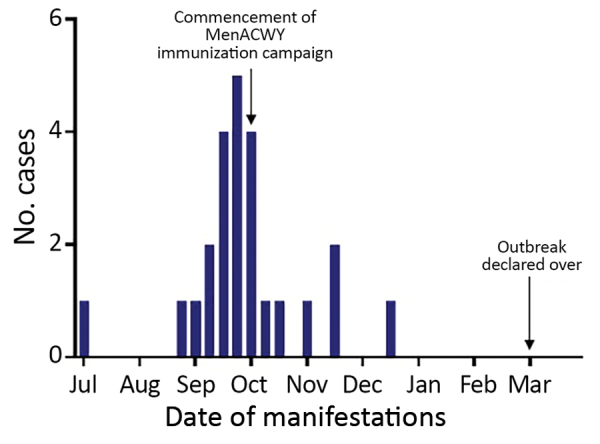
Timeline for outbreak of meningococcal W disease, showing case manifestations, by month, Central Australia, 2017. MenACWY, quadrivalent meningococcal A, C, W, Y conjugate vaccine.

During the 5 months starting in July 2017, a total of 24 patients with MenW were admitted to the Alice Springs Hospital pediatric ward. We found an attack rate of 10.9 cases/100,000 persons for the ATSI population <15 years of age (Figure 2). In comparison, in Australia during 2011–2015, there were 0–2 cases/year in the ATSI population (0–0.9 cases/100,000 persons/year) and 9 cases during 2016 (4.1 cases/100,000 persons) (*20*,*25*,*32*).

During the 9-month outbreak period, ≈649 patients were managed under the fever protocol. In addition to the 24 cases of MenW, 1 case was caused by meningococcal serogroup Y and 1 by nongroupable meningococci. One additional case-patient had MenW during March 2017 (isolated meningococcemia) but the isolate was a different sequence type (ST) and was not classified as part of the outbreak. Two additional cases of MenW occurred in adult patients during this time but were not managed according to the pediatric fever protocol.

The 24 cases of MenW during the outbreak comprised 23 cases of IMD and 1 case of MenW conjunctivitis. Only 1 patient had had possible contact with an index case-patient; this contact was 4 days before development of MenW infection. For all other patients, there was no identified contact with an index case-patient.

We compiled demographic and clinical details for the 24 outbreak patients (Table 2). The patients came from 18 of the 58 communities in the Alice Springs Hospital catchment area (Figure 1). All cases were among ATSI children. Fifteen (63%) patients were transferred to Alice Springs Hospital from remote communities by the RFDS. There were more male (71%) patients than female (29%) patients. Patients ranged in age from 3 months to 14 years; 54% were <4 years of age.

**Table 2:** Characteristics of 24 case-patients who had meningococcal serogroup W infection, Central Australia, 2017*

Characteristic	Value
Age, y	
Median (25%–75% percentile)	5.2 (1.7–7.2)
Range	0.3–14.7
Sex	
M	17 (71)
F	7 (29)
Australian Aboriginal	24 (100)
No. communities affected (n = 58)	18 (31)
Location of communities	
Northern Territory	14 (78)
South Australia	3 (17)
Western Australia	1 (5)
Air retrieval to Alice Springs Hospital	15 (63)
Signs/symptoms	
Fever	23 (96)
Tachycardia	18 (75)
Vomiting	10 (42)
Cough/respiratory distress	7 (29)
Arthritis/arthralgia	8 (33)
Meningism	7 (29)
Hypotension	6 (25)
Purpuric rash	3 (13)
Diarrhea	2 (8)
Petechial rash	1 (4)
Conjunctivitis	1 (4)
Diagnosis	
Isolated meningococcemia	12 (50)
Meningitis	6 (24)
Bacteremic pneumonia	4 (16)
Septic arthritis	1 (4)
Conjunctivitis	1 (4)
Admission location	
Pediatric ward	20 (83)
Intensive care unit	4 (17)
Length of admission, d	
Median (25%–75% percentile)	5.5 (5–8.5)
Range	4–128
Duration of antimicrobial drug use, d	
Median (25%–75% percentile)	6 (5–8)
Range	4–14
Time from visit to health service to first dose of ceftriaxone, h	
Median (25%–75% percentile)	4.7 (1.6–15.6)
Range	0.6–54.9
Complications	
Persistent bradycardia	4 (17)
Persistent arthralgia	4 (17)
Persistent fevers	3 (13)
Coagulopathy	1 (4)
Acute kidney injury	1 (4)
Below-knee amputation	1 (4)
Culture positive	17 (71)
Penicillin sensitivity	
Intermediate	15 (88)
Resistant	2 (12)
PCR positive	23 (96)

*Values are no. (%) unless otherwise indicated.

The MenACWY immunization campaign started in early October 2017. After mid-October, 6 additional cases of IMD were reported; the final case-patient came to the hospital on December 23, 2017 (Figure 2). All cases of MenW occurred in unimmunized patients. One patient with suspected MenW infection had received the MenACWY vaccine 12 days before being identified. For this case-patient, the blood PCR result was equivocal; the patient was given intravenous antimicrobial drugs for 5 days, but results for this patient are not included in this report.

Seventeen (71%) cases were confirmed by culture (blood, synovial fluid, or eye swab specimen) and 7 by PCR (blood or CSF). Of the cases that were culture positive, susceptibility to penicillin was intermediate (MIC 0.064 to <0.5 μg/mL) for 15 cases (88%) and resistant (MIC >0.5 to 1 μg/mL) for 2 cases (12%). No isolates were penicillin susceptible, and all were susceptible to ceftriaxone. Multilocus sequence typing of isolates indicated that all belonged to clonal complex 11 (MenW:cc11), ST 1287 (*33*).

All but 1 case-patient (96%) had samples that were tested by PCR. For 7 case-patients, this testing was necessary to obtain a diagnosis because ceftriaxone had been administered before blood or CSF collection. For the remaining 16 case-patients, PCR testing was used to obtain a more rapid diagnosis because PCR results were available in a shorter time frame than culture results, thereby enabling prompt allocation of appropriate resources.

All case-patients with invasive disease had a fever (temperature >38°C) (Table 2). The time interval between first arrival at a clinic and commencement of appropriate antimicrobial drug treatment (ceftriaxone) varied between 36 minutes and almost 55 hours (median 4.7 hours) (Table 2). For the patient who received ceftriaxone 55 hours after first arrival, the patient received daily examinations at the local clinic for respiratory symptoms. The first documented fever was on day 3 of illness, at which point ceftriaxone was administered and the patient was transferred to Alice Springs Hospital by RFDS. The patient was given a diagnosis of isolated meningococcemia, given ceftriaxone for 5 days, and showed a full recovery.

Seven patients received penicillin (amoxicillin or benzathine penicillin) before ceftriaxone. Of those patients, 5 had positive cultures, 4 had intermediate penicillin sensitivity, and 1 had penicillin resistance. For 6 case-patients, penicillin was administered before specimen collection (≈8–24 hours before for each case-patient). The mean time from initial clinic arrival to first dose of ceftriaxone was significantly longer for these patients compared with patients who received ceftriaxone as first-line treatment (26.1 hours vs. 6.0 hours; p = 0.0011). The mean length of stay was also significantly longer for patients who initially received penicillin compared with patients who received ceftriaxone as first-line therapy (25.6 days vs. 6.4 days, p = 0.06). These patients include 1 patient who required transfer to a tertiary center because of meningococcemia, purpura fulminans, and a below-knee amputation and who had a prolonged hospital admission (128 days). The time interval from initial clinic arrival to the first dose of ceftriaxone for this patient was 16.8 hours. Benzathine penicillin had been administered previously, and the isolate from this case-patient showed intermediate susceptibility to penicillin.

There were no deaths during the outbreak. Four (17%) patients (2 with meningitis, 1 with isolated meningococcemia, and 1 with purpura fulminans) were initially admitted to the ICU; the remainder were admitted directly to the pediatric ward. When we excluded the patient who had major illness and required transfer to a tertiary center, we found that the median length of stay for the remaining 23 patients was 5 days (range 4–15 days) (Table 2).

The most common diagnosis was isolated meningococcemia (50%), followed by meningitis (25%), bacteremic pneumonia (17%), septic arthritis (4%), and conjunctivitis (4%) (Table 2). Typical manifestations occurred across all age groups, whereas atypical manifestations occurred only in younger patients (<7 years of age). Of the 12 patients with isolated meningococcemia, 7 (58%) were suspected by the admitting clinician of having alternative dignoses: pneumonitis/bronchiolitis (2), gastroenteritis (2), meningitis (1), acute rheumatic fever (1), and viral illness (1). Three patients had a purpuric rash (purpura fulminans developed in 1 of these patients). There was no significant difference in age (mean 9.3 years vs. 3.8 years; p = 0.08) or the time interval to receiving ceftriaxone (mean 11.5 hours vs. 11.5 hours; p = 0.30) between patients with isolated meningococcemia who had a purpuric rash compared with patients who did not have this rash.

Six (25%) patients had meningitis, all confirmed by PCR for CSF. One patient who had meningitis also had meningococcemia (positive blood culture and PCR). Four patients with meningitis (80%) had persistent bradycardia during their admission.

Four (17%) patients had MenW bacteremic pneumonia. Eight (33%) patients had arthritis or arthralgia as an initial symptom, but only 1 patient was given a diagnosis of MenW septic arthritis. One patient had severe, bilateral MenW conjunctivitis that was confirmed by culture and PCR. Although conjunctivitis was not invasive disease, this patient was admitted for administration of parenteral antimicrobial drugs and required a public health response. Despite appropriate antimicrobial treatment, 4 patients had a prolonged admission at Alice Springs Hospital (12–15 days) because of persistent fevers, persistent arthralgias, or both.

Anaphylaxis developed in 1 patient managed under the fever protocol after administration of ceftriaxone. This patient was given treatment in the emergency department at Alice Springs Hospital and admitted to the ICU overnight for observation. The patient had had no history of allergy or anaphylaxis to any antimicrobial drug. This episode was the only adverse event that occurred as a result of the fever protocol.

## Discussion

The outbreak of MenW among the ATSI population in Central Australia was unique because it juxtaposed a well-resourced healthcare system against challenges related to covering vast distances, a socially disadvantaged population, and a disease process that was rapid and unpredictable. The clinical and public health responses resulted in no deaths and only 1 case-patient who had major illness, in contrast to previously documented case-fatality rates elsewhere of 10% (*2*,*6*,*34*,*35*).

The ATSI population of Australia has a higher rate of IMD than the non-Indigenous population (*36*,*37*). During 2016, Indigenous persons in Australia comprised 3.3% of the total population of this country but accounted for >10% of IMD reports (*38*). The ATSI population has a high disease burden for numerous infectious conditions, including lower respiratory tract infections, group A *Streptococcus* skin sores, and scabies (*39*,*40*). The underlying determinants relate to socioeconomic factors and include higher rates of overcrowded housing, educational disadvantage, poorer nutrition, higher unemployment rates, and reduced access to specialist medical care (*41*). These factors overlap with known risk factors for IMD (*42*–*44*).

Early signs of IMD make it difficult to distinguish from other causes of febrile illness. Therefore, the broad case definition used in the fever protocol was considered necessary, given the remote location of the affected communities. The spread of a relatively small number of pediatric patients across a wide and remote area, with access to well-equipped local medical services, an air retrieval service (RFDS), and a secondary pediatric referral service, made the fever protocol response possible.

A total of 25% of case-patients had atypical manifestations of IMD (bacteremic pneumonia, septic arthritis, conjunctivitis), and all those cases were in younger children (<7 years of age). In previous MenW outbreaks overseas, rates of atypical manifestations have varied from 4% to 25% (*29*,*45*,*46*). Gastrointestinal symptoms (vomiting, diarrhea, or both) were present in 11 (44%) patients during this outbreak, although only 2 (8%) patients were initially believed to have gastroenteritis at initial clinic visit. Gastrointestinal symptoms have been associated with a high case-fatality rate in 2 previous studies, although these studies involved a hypervirulent ST11 strain of MenW (*8*,*46*).

For the 17 case-patients from whom MenW was isolated by culture, reduced susceptibility to penicillin reflected previously documented antimicrobial drug susceptibility patterns for MenW in Australia (*22*,*47*). ST1287 isolates have previously been described in sporadic cases in Western Australia and were associated with penicillin resistance (*48*). Of the 7 patients in this outbreak who initially received penicillin, 1 patient had a major illness (purpura fulminans) and a complication (below-knee amputation). The other 6 patients did not have a complicated clinical course. Treatment with penicillin is still effective against penicillin-intermediate strains if given at high doses (*48*), and it is possible that the clinical course for these patients was somewhat attenuated compared with if they had received no initial treatment before being given ceftriaxone.

Prolonged hospital admission was required for 5 case-patients (4 patients at Alice Springs Hospital and 1 patient at a tertiary referral center). The cause of the prolonged duration of symptoms for the 4 patients at Alice Springs Hospital was not clear, but all patients had their illnesses resolve before discharge from the hospital. No characteristics clearly differentiated these patients from the rest of the cohort. The 4 patients had meningitis, septic arthritis, bacteremic pneumonia, and meningococcemia. Two of these patients had a comparatively longer time interval between first presentation to a clinic and receiving their first dose of ceftriaxone (20.8 hours for the patient with meningitis and 39.2 hours for the patient with septic arthritis). However, the intervals were much shorter for the other 2 patients (1.2 hours for the patient with bacteremic pneumonia and 14.3 hours for the patient with meningococcemia). For the second 2 patients, there was an interval of only 2.2 hours between their first documented fever and their first dose of ceftriaxone. These case-patients were clinically challenging, which resulted in longer admissions, additional investigations (e.g., echocardiogram, lumbar puncture, joint aspirate) and longer courses of antimicrobial drug therapy. A possible cause was immune complex disease, known to occur in the subacute phase of meningococcal disease. This disease can cause symptoms of arthritis, vasculitis, pleuritis, body temperature increase, and increased levels of inflammatory markers 4–10 days after systemic disease (*49*).

The public health response coordinated by CDC required complex planning because of geographic and logistical challenges. This response was further limited by movement of persons between communities, which is inherent to the ATSI population of Central Australia. This limitation complicated the task of locating contacts for provision of prophylactic antimicrobial drugs, as well as the subsequent MenACWY vaccination campaign.

­The number of pediatric meningococcal case-patients admitted to Alice Springs Hospital decreased after the vaccination campaign was initiated (Figure 2). The fever protocol considered persons to be protected 4 weeks after vaccination (*50*), at which time their management was based on clinical manifestations, rather than on the presumption of IMD.

The response incurred costs and might not be practical for other settings. The response also greatly increased the workload of the local medical clinics, the RFDS, and the Alice Springs Hospital emergency department and pediatric department. There was an economic impact of increased staffing; additional investigations, including meningococcal PCR and blood cultures; and increased provision of antimicrobial drugs. However, this impact must be balanced against the cost of the probable increased rate of complications and transfers to a tertiary center, which might have occurred if treatment had been delayed. The empiric administration of ceftriaxone to all patients who had fever during the early phase of the outbreak also carries the potential for promoting antimicrobial drug resistance.

Replicating the response to this outbreak in more densely populated areas would be challenging, and a different approach might be appropriate in situations in which direct access to secondary and tertiary health services is available. However, the response to this outbreak was extremely effective in this particular setting and resulted in a low illness rate and a zero fatality rate for a disease that is typically devastating.

## References

[1] Borrow R, Alarcón P, Carlos J, Caugant DA, Christensen H, Debbag R, et al.; Global Meningococcal Initiative. The Global Meningococcal Initiative: global epidemiology, the impact of vaccines on meningococcal disease and the importance of herd protection. Expert Rev Vaccines. 2017;16:313–28. 10.1080/14760584.2017.1258308 27820969

[2] Manchanda V, Gupta S, Bhalla P. Meningococcal disease: history, epidemiology, pathogenesis, clinical manifestations, diagnosis, antimicrobial susceptibility and prevention. Indian J Med Microbiol. 2006;24:7–19. 10.4103/0255-0857.19888 16505549

[3] Veitch MG, Owen RL. Rise in invasive serogroup W meningococcal disease in Australia 2013-2015. Commun Dis Intell Q Rep. 2016;40:E451–3. 28043218 10.33321/cdi.2016.40.49

[4] Lewis LA, Ram S. Meningococcal disease and the complement system. Virulence. 2014;5:98–126. 10.4161/viru.26515 24104403 PMC3916388

[5] Thompson MJ, Ninis N, Perera R, Mayon-White R, Phillips C, Bailey L, et al. Clinical recognition of meningococcal disease in children and adolescents. Lancet. 2006;367:397–403. 10.1016/S0140-6736(06)67932-4 16458763

[6] Martin NV, Ong KS, Howden BP, Lahra MM, Lambert SB, Beard FH, et al.; Communicable Diseases Network Australia MenW Working Group. Rise in invasive serogroup W meningococcal disease in Australia 2013-2015. Commun Dis Intell Q Rep. 2016;40:E454–9. 28043219 10.33321/cdi.2016.40.50

[7] Vienne P, Ducos-Galand M, Guiyoule A, Pires R, Giorgini D, Taha M-K, et al. The role of particular strains of *Neisseria meningitidis* in meningococcal arthritis, pericarditis, and pneumonia. Clin Infect Dis. 2003;37:1639–42. 10.1086/379719 14689345

[8] Campbell H, Parikh SR, Borrow R, Kaczmarski E, Ramsay ME, Ladhani SN. Presentation with gastrointestinal symptoms and high case fatality associated with group W meningococcal disease (MenW) in teenagers, England, July 2015 to January 2016. Euro Surveill. 2016;21:1–4. 10.2807/1560-7917.ES.2016.21.12.30175 27035055

[9] Schaad UB. Arthritis in disease due to *Neisseria meningitidis.* Rev Infect Dis. 1980;2:880–8. 10.1093/clinids/2.6.880 7012989

[10] Barquet N, Gasser I, Domingo P, Moraga FA, Macaya A, Elcuaz R. Primary meningococcal conjunctivitis: report of 21 patients and review. Rev Infect Dis. 1990;12:838–47. 10.1093/clinids/12.5.838 2237127

[11] Orden B, Martínez R, Millán R, Belloso M, Pérez N. Primary meningococcal conjunctivitis. Clin Microbiol Infect. 2003;9:1245–7. 10.1111/j.1469-0691.2003.00799.x 14686993

[12] Lingappa JR, Al-Rabeah AM, Hajjeh R, Mustafa T, Fatani A, Al-Bassam T, et al. Serogroup W-135 meningococcal disease during the Hajj, 2000. Emerg Infect Dis. 2003;9:665–71. 10.3201/eid0906.020565 12781005 PMC3000138

[13] Yezli S, Assiri AM, Alhakeem RF, Turkistani AM, Alotaibi B. Meningococcal disease during the Hajj and Umrah mass gatherings. Int J Infect Dis. 2016;47:60–4. 10.1016/j.ijid.2016.04.007 27062987

[14] Ladhani SN, Ramsay M, Borrow R, Riordan A, Watson JM, Pollard AJ. Enter B and W: two new meningococcal vaccine programmes launched. Arch Dis Child. 2016;101:91–5. 10.1136/archdischild-2015-308928 26672098 PMC4717420

[15] Smith-Palmer A, Oates K, Webster D, Taylor S, Scott KJ, Smith G, et al. IMT and investigation team in Sweden. Outbreak of *Neisseria meningitidis* capsular group W among scouts returning from the World Scout Jamboree, Japan, 2015. Euro Surveill. 2016;21:1–7. 10.2807/1560-7917.ES.2016.21.45.30392 PMC5144938 27918267

[16] Nathan N, Rose AMC, Legros D, Tiendrebeogo SRM, Bachy C, Bjørløw E, et al. Meningitis serogroup W135 outbreak, Burkina Faso, 2002. Emerg Infect Dis. 2007;13:920–3. 10.3201/eid1306.060940 17553237 PMC2792856

[17] von Gottberg A, du Plessis M, Cohen C, Prentice E, Schrag S, de Gouveia L, et al.; Group for Enteric, Respiratory and Meningeal Disease Surveillance in South Africa. Emergence of endemic serogroup W135 meningococcal disease associated with a high mortality rate in South Africa. Clin Infect Dis. 2008;46:377–86. 10.1086/525260 18181736

[18] Abad R, López EL, Debbag R, Vázquez JA. Serogroup W meningococcal disease: global spread and current affect on the Southern Cone in Latin America. Epidemiol Infect. 2014;142:2461–70. 10.1017/S0950268814001149 24831052 PMC9151320

[19] Campbell H, Saliba V, Borrow R, Ramsay M, Ladhani SN. Targeted vaccination of teenagers following continued rapid endemic expansion of a single meningococcal group W clone (sequence type 11 clonal complex), United Kingdom 2015. Euro Surveill. 2015;20:21188. 10.2807/1560-7917.ES2015.20.28.21188 26212140

[20] Australian Government Department of Health. Invasive meningococcal disease national surveillance report, December 2017 [cited 2018 Jun 27]. http://www.health.gov.au/internet/main/publishing.nsf/Content/5FEABC4B495BDEC1CA25807D001327FA/$File/1Jan-31-Dec2017-Consol-Invasive-Men-W.pdf

[21] National Centre for Immunisation Research and Surveillance. History of vaccination in Australia, December 2017 [cited 2018 Jun 18]. http://www.ncirs.org.au/sites/default/files/2018-12/Meningococcal-history-Dec-2018.pdf

[22] Dolan Thomas J, Hatcher CP, Satterfield DA, Theodore MJ, Bach MC, Linscott KB, et al. sodC-based real-time PCR for detection of *Neisseria meningitidis.* PLoS One. 2011;6:e19361. 10.1371/journal.pone.0019361 21573213 PMC3088665

[23] Jordens JZ, Heckels JE. A novel porA-based real-time PCR for detection of meningococcal carriage. J Med Microbiol. 2005;54:463–6. 10.1099/jmm.0.45847-0 15824424

[24] Jolley KA, Brehony C, Maiden MC. Molecular typing of meningococci: recommendations for target choice and nomenclature. FEMS Microbiol Rev. 2007;31:89–96. 10.1111/j.1574-6976.2006.00057.x 17168996

[25] Australian Bureau of Statistics. 2016 Census: Aboriginal and/or Torres Strait Islander peoples QuickStats [cited 2019 Aug 16]. https://quickstats.censusdata.abs.gov.au/census_services/getproduct/census/2016/quickstat/IQS036

[26] Vossen M, Mitteregger D, Steininger C. Meningococcal pneumonia. Vaccine. 2016;34:4364–70. 10.1016/j.vaccine.2016.07.013 27443594

[27] Romero-Gomez MP, Rentero Z, Paño JR, Mingorance J. Bacteraemic pneumonia caused by *Neisseria meningitidis* serogroup Y. Respir Med Case Rep. 2012;5:23–4. 10.1016/j.rmedc.2011.11.005 26057210 PMC3920377

[28] Centre for Disease Control. Meningococcal disease: signs and symptoms [cited 2019 Aug 22]. https://www.cdc.gov/meningococcal/about/symptoms.html

[29] Gaschignard J, Levy C, Deghmane AE, Dubos F, Muszlak M, Cohen R, et al. Invasive serogroup w meningococcal disease in children: a national survey from 2001 to 2008 in France. Pediatr Infect Dis J. 2013;32:798–800. 10.1097/INF.0b013e31828e9e91 23838782

[30] Communicable Diseases Network Australia. Invasive meningococcal disease CDNA national guidelines for public health units, 2017 [cited 2019 Aug 22]. https://www1.health.gov.au/internet/main/publishing.nsf/Content/0A31EEC4953B7E6FCA257DA3000D19DD/$File/IMD-SoNG.pdf

[31] The Northern Territory Department of Health. Brief for the meningococcal outbreak task force. Darwin: The Department; 2018.

[32] Australian Government Department of Health. Meningococcal disease (invasive) public dataset. National Notifiable Diseases Surveillance System [cited 2019 Aug 13]. http://www9.health.gov.au/cda/source/pub_menin.cfm

[33] PubMLST. *Neisseria* profile/sequence definitions database [cited 2019 Aug 15]. https://pubmlst.org/bigsdb?db=pubmlst_neisseria_seqdef

[34] Rosenstein NE, Perkins BA, Stephens DS, Lefkowitz L, Cartter ML, Danila R, et al. The changing epidemiology of meningococcal disease in the United States, 1992-1996. J Infect Dis. 1999;180:1894–901. 10.1086/315158 10558946

[35] Rosenstein NE, Perkins BA, Stephens DS, Popovic T, Hughes JM. Meningococcal disease. N Engl J Med. 2001;344:1378–88. 10.1056/NEJM200105033441807 11333996

[36] Harley D, Hanna JN, Hills SL, Bates JR, Smith HV. Epidemiology of invasive meningococcal disease in north Queensland, 1995 to 1999. Commun Dis Intell Q Rep. 2002;26:44–50. 11950202 10.33321/cdi.2002.26.9

[37] Massey P, Durrheim D. Aboriginal and Torres Strait Islander peoples at higher risk of invasive meningococcal disease in NSW. N S W Public Health Bull. 2008;19:100–3. 10.1071/NB07047 18638436

[38] Australian Government. Australian Institute of Health and Welfare. Vaccine preventable disease among Aboriginal and Torres Strait Islander people [cited 2019 Aug 22]. https://www.aihw.gov.au/getmedia/2fca3ed6-d242-4454-a00f-e298dd120ccb/aihw-phe-236_ATSI.pdf.aspx

[39] Cuningham W, McVernon J, Lydeamore MJ, Andrews RM, Carapetis J, Kearns T, et al. High burden of infectious disease and antibiotic use in early life in Australian Aboriginal communities. Aust N Z J Public Health. 2019;43:149–55. 10.1111/1753-6405.12876 30727032

[40] Hendrickx D, Bowen AC, Marsh JA, Carapetis JR, Walker R. Ascertaining infectious disease burden through primary care clinic attendance among young Aboriginal children living in four remote communities in Western Australia. PLoS One. 2018;13:e0203684. 10.1371/journal.pone.0203684 30222765 PMC6141079

[41] Parnaby MG, Carapetis JR. Rheumatic fever in indigenous Australian children. J Paediatr Child Health. 2010;46:527–33. 10.1111/j.1440-1754.2010.01841.x 20854325

[42] Bilukha OO, Rosenstein N; National Center for Infectious Diseases, Centers for Disease Control and Prevention (CDC). Prevention and control of meningococcal disease. Recommendations of the Advisory Committee on Immunization Practices (ACIP). MMWR Recomm Rep. 2005;54(RR-7):1–21. 15917737

[43] Umaru ET, Ludin AN, Majid MR, Sabri S, Moses C, Enegbuma W, et al. Risk factors responsible for the spread of meningococcal meningitis. Int J Educ Res. 2013;1:1–30.

[44] Olea A, Matute I, González C, Delgado I, Poffald L, Pedroni E, et al. Case–control study of risk factors for meningococcal disease in Chile. Emerg Infect Dis. 2017;23:1070–8. 10.3201/eid2307.160129 28628448 PMC5512488

[45] Ladhani SN, Beebeejaun K, Lucidarme J, Campbell H, Gray S, Kaczmarski E, et al. Increase in endemic *Neisseria meningitidis* capsular group W sequence type 11 complex associated with severe invasive disease in England and Wales. Clin Infect Dis. 2015;60:578–85. 10.1093/cid/ciu881 25389259

[46] Moreno G, López D, Vergara N, Gallegos D, Advis MF, Loayza S. [Clinical characterization of cases with meningococcal disease by W135 group in Chile, 2012] [in Spanish]. Rev Chilena Infectol. 2013;30:350–60. 24248103 10.4067/S0716-10182013000400002

[47] Lahra MM, Enriquez R. Australian meningococcal surveillance programme annual report, 2016. Commun Dis Intell Q Rep. 2017;41:E369–82. 29864389 10.33321/cdi.2017.41.46

[48] Mowlaboccus S, Jolley KA, Bray JE, Pang S, Lee YT, Bew JD, et al. Clonal expansion of new penicillin-resistant clade of *Neisseria meningitidis* serogroup W clonal complex 11, Australia. Emerg Infect Dis. 2017;23:1364–7. 10.3201/eid2308.170259 28609259 PMC5547816

[49] Goedvolk CA, von Rosenstiel IA, Bos AP. Immune complex associated complications in the subacute phase of meningococcal disease: incidence and literature review. Arch Dis Child. 2003;88:927–30. 10.1136/adc.88.10.927 14500317 PMC1719308

[50] Sanofi-Aventis Pte Ltd. Menactra: meningococcal (groups A,C,Y and W-135) polysaccharide diptheria toxoid conjugate vaccine Australian product information, 2011 [cited 2020 Mar 31]. https://www.menactra.com

